# FA-nf: A Functional Annotation Pipeline for Proteins from Non-Model Organisms Implemented in Nextflow

**DOI:** 10.3390/genes12101645

**Published:** 2021-10-19

**Authors:** Anna Vlasova, Toni Hermoso Pulido, Francisco Camara, Julia Ponomarenko, Roderic Guigó

**Affiliations:** 1 Barcelona Supercomputing Centre (BSC-CNS), Jordi Girona, 29, 08034 Barcelona, Spain; anna.vlasova@bsc.es; 2 Institute for Research in Biomedicine (IRB Barcelona), The Barcelona Institute of Science and Technology, Baldiri Reixac, 10, 08028 Barcelona, Spain; 3 Centre for Genomic Regulation (CRG), The Barcelona Institute for Science and Technology, Dr. Aiguader 88, 08003 Barcelona, Spain; francisco.camara@crg.eu (F.C.); julia.ponomarenko@crg.eu (J.P.); roderic.guigo@crg.cat (R.G.); 4 Universitat Pompeu Fabra (UPF), 08003 Barcelona, Spain

**Keywords:** functional annotation, containerization, pipeline, reproducibility

## Abstract

Functional annotation allows adding biologically relevant information to predicted features in genomic sequences, and it is, therefore, an important procedure of any de novo genome sequencing project. It is also useful for proofreading and improving gene structural annotation. Here, we introduce FA-nf, a pipeline implemented in Nextflow, a versatile computational workflow management engine. The pipeline integrates different annotation approaches, such as NCBI BLAST+, DIAMOND, InterProScan, and KEGG. It starts from a protein sequence FASTA file and, optionally, a structural annotation file in GFF format, and produces several files, such as GO assignments, output summaries of the abovementioned programs and final annotation reports. The pipeline can be broken easily into smaller processes for the purpose of parallelization and easily deployed in a Linux computational environment, thanks to software containerization, thus helping to ensure full reproducibility.

## 1. Introduction

The recent development of sequencing technologies allows researchers to obtain fast and relatively cheap assemblies of the genomes of many organisms, which has led to a significant increase in available genomes from all kingdoms of life [1,2]. However, the assembly of the genome and its structural gene annotation (the gene and transcript map) is just an initial step in answering some biological questions. Yet, annotated features represent only a list with coordinates in the genome and its corresponding sequence with some abbreviation instead of the name, in particular, for the de novo gene annotation. To add biologically meaningful value to those features, such as putative function, presence of specific domains, cellular localization, metabolic pathways, GO terms, and gene descriptions, the researcher needs to perform so-called functional annotation [3]. If features are predicted protein coding genes, their function can be automatically assigned, based on sequence and/or 3D structural similarity to proteins in available databases.

**Figure 1:**
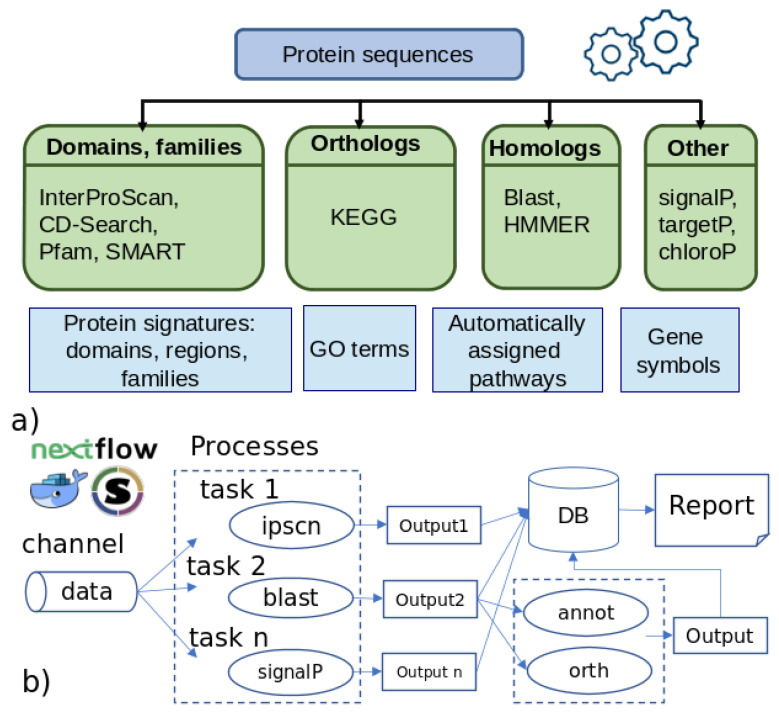
(**a**) A typical functional annotation workflow; (**b**) simplified flowchart of FA-nf pipeline.

The typical functional annotation workflow involves combining different methods that analyze a protein sequence from different angles and integrating results into a consensus annotation (Figure 1a). The simplest approach to an annotation relies on performing a homology search against a set of representative sequences, such as a BLAST search against the UniProt or NCBI NR (non-redundant) protein databases [4,5,6]. Because the sequence similarity might occur between two evolutionarily unrelated sequences, due to the presence of common domains [7], it is also essential to assign a predicted protein to one of the known orthologous groups and infer functional annotation from these orthologs [8,9,10]. Another approach implies finding known protein signatures by using diagnostic models, such as hidden Markov models (HMM), or searching for regular expressions against specialized databases, such as InterPro [11], which in turn, contains PANTHER [12], Pfam [13], and SUPERFAMILY [14] databases, among many others.

There are already existing frameworks for the functional annotation of genomes. Some of them are accessible online with an easy-to-use web interface [9,15,16,17], while others require a local installation [16,18,19,20] (Table 1). Tools that are solely available online can have a limitation on the number of sequences that can be annotated in one run, and therefore, cannot always be used for high-throughput analysis. On the other hand, those pipelines that can be run locally often require specialized computational knowledge, as they depend on the installation of external programs and databases. Furthermore, it may also be important to optimize parallelization processes for speeding up the annotation.

Here, we present a scalable and parallelizable workflow for functional annotation, named FA-nf, implemented in the Nextflow framework [21] with Docker container images generated for every process. The pipeline requires, as input, protein sequences in the standard FASTA format and, optionally, a gene structural annotation encoded in GFF3 format. The output of the pipeline consists of several annotation files in the plain text format, with a consensus annotation in GFF3 format and a final HTML report. The results from the different processes are stored in a relational database upon process completion and retrieved at the end of the annotation process. The FA-nf pipeline integrates tools that are widely used in the community and includes all important steps of protein annotation. Importantly, the Nextflow framework also avoids the problem of installing software dependencies by the user and can, therefore, be used by researchers with less bioinformatic expertise [22] than is required by other existing pipelines (Figure 1).

## 2. Materials and Methods

### 2.1. Overview of the Pipeline

The usage of a workflow management engine can simplify the development and maintenance of pipelines [23]. During recent years, the bioinformatics community has been progressively adopting various management systems, such as Snakemake [24], Galaxy [25], Nextflow [21]. For the FA-nf pipeline, we selected Nextflow because it has provided support for container technologies, different HPC queue schedulers and cloud providers from its inception. This eases the portability to different environments and has already become the choice of both specific and wide-reach projects [26,27]. 

Every Nextflow process (that is, a pipeline step) is associated with a specific container image. Whenever possible, if common software (such as NCBI Blast+ [28]) is already available and fits our requirements, container images from official sources and Biocontainers.pro initiative are preferred [29]. Otherwise, if images are either modified in order to work with the pipeline (e.g., with InterProscan) or based on software that does not comply with Open Source Initiative licenses [30] (e.g., with SignalP [31]), associated recipes are published in a public Git repository [32]. This way, container images can be generated in advance and placed in a suitable location of the HPC environment, where the pipeline is executed.

Some steps in the pipeline consist of scripts for processing, gathering and merging the outputs of the different applications involved (e.g., NCBI BLAST+ or Interproscan [33]), executed in either parallel or preceding steps. These are mostly programmed in Perl and kept in a specific and separate container in order to warrant code reproducibility.

**Figure 2:**
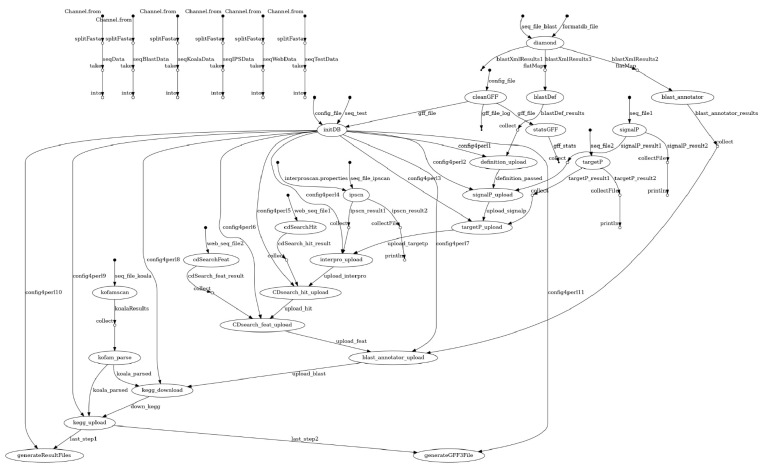
Automatically generated schema from Nextflow output.

Data from the different analyses steps are retrieved and stored in a relational database to simplify the gathering of information and predictions for every protein entry (Figure 1b and Figure 2). This also substantially speeds up the generation of statistics reports and final results. Support for two relational database management systems is provided: SQLite and MySQL/MariaDB. The former, which stores the database in a single file, requires little initial configuration from the user side and allows major portability. However, SQLite is far less performant in environments where shared file systems are involved (quite common in most HPC setups) [34]. For that latter case, the MySQL/MariaDB option is provided. Users need to point to an accessible database server instance that will receive data uploads from the ongoing processes. For the sake of convenience, a wrapper script that launches a MariaDB Singularity container instance is also provided [35]. At the time of writing, this was tested on the SGE-compatible queue systems and thus, can be easily adapted to other systems as far as proper intercommunication among the involved nodes is warranted.

The FA-nf pipeline also allows to fine-tune the resources assigned to its different processes. Within a configuration file, customarily named nextflow.config, as long as processes are properly labelled, the user can adjust the minimum assigned CPU cores and available memory to be demanded from a computation node. Likewise, for the node queue, the user can specify where to submit the processes and the maximum amount of time each process is allowed to be kept running in the node. Additionally, with FA-nf, it is possible to specify (in params.config file) the number of sequences (chunk size) that are going to be processed, depending on the type of process.

The pipeline is defined by default in a debug mode with a limited number of chunks to be processed, which can also be specified with a debugSize parameter. 

By adjusting the different size-related parameters in the params.config file, and taking into consideration the number, but also the length, of the analyzed protein sequences, the underlying HPC infrastructure (e.g., batch queue systems, number of nodes, long/short queues or disk access) and the methods used in the process (e.g., web services vs local applications), the user can improve the performance and reduce the execution time of the entire annotation process. Once parameters are proven to be good enough, they can be reused in other annotation efforts using the same methods with genomes of similar sizes under the same computational environment.

### 2.2. Preprocessing

The minimum input of the pipeline is a FASTA file with protein sequences. A GFF file, either retrieved from public resources or from a gene structural annotation workflow, is normally expected as well. If a GFF file is provided, the same protein IDs in the FASTA file should be used in the corresponding entries of the GFF.

Despite existing GFF3 recommendations, there is a large diversity of GFF formats. In order to ensure that input GFF files can go through downstream processes and no prediction is lost, an initial automatic verification and cleaning step with AGAT Toolkit is introduced [36]. Common GFF3 formats retrieved from ENSEMBL, NCBI and also generated with PASA and TransDecoder pipelines are successfully tested [37,38].

Once this previous verification is performed, entries from GFF and FASTA files are further checked and imported into a database, which will act as a central reference point for the upstream processes. 

### 2.3. Analysis

Once protein sequence IDs are recorded in the central FA-nf database, their sequences are grouped in chunks and distributed in different parallel computation processes. This stage represents the main bulk of the pipeline, and it is actually the one that benefits the most from using a workflow engine and the HPC infrastructure. We discuss some of the involved applications below.

### 2.4. NCBI BLAST+ and DIAMOND as Annotation Sources

At the time of writing, users could choose either NCBI BLAST+ or DIAMOND [39] as annotation sources based on the sequence homology. As a rule of thumb, when using the same FA-nf parameters, NCBI BLAST+ provides slightly more potential results from hits, but with substantially more computation time. In that case, when NCBI BLAST+ is used, it is preferable to run workload tasks that contain fewer sequences than when DIAMOND is used.

Instead of a BLAST2GO file, the BLAST annotator service is available as a downstream step from the processes above. This is used for retrieving GO correspondences extracted from the highest hits of the sequence homology results. However, for using it, a web service accessible by the pipeline needs to be set up in advance. In our case, this web service is fed by using a database populated by the UniProt to GO (GOA) correspondences [40] and UniProt Idmapping file [5]. Therefore, even if the used BLAST database is not made of UniProt entries (e.g., of NCBI GenBank sequences), as long as the Idmapping file contains correspondences to actual UniProt entries, the database can be safely used. If suitable input files are employed, different BLAST annotator services could also be designed, using other sources instead.

The service uses the hits that fit the given Blast e-value threshold and provides different GO retrieval approaches. By default, it uses a “common” mode, which, in a conservative fashion, retrieves only those GO codes that are common in all selected hits. Alternatively, at the time of writing, we could choose an “all” mode, that retrieves all available GO codes from the hits, or, instead, a “most” mode, which picks up GO codes if present in more than half of the hits.

### 2.5. KAAS and KOFAM

In order to take advantage of the capacity of annotation from KEGG from which, so far, we retrieve notably KO (KEGG orthology) codes and their GO codes correspondences, it is possible to use either the KAAS web service [41] or the KofamKOALA standalone program [42]. In the first case, the output file of KAAS service, which needs to be manually retrieved in advance, must be provided as a parameter (keggFile) in the config file. This KAAS output file must be generated from the same input FASTA file that is used in the pipeline. Alternatively, if no file generated from KAAS is provided, KofamKOALA will be used to generate a similar file that also contains sequence IDs matching KO codes.

### 2.6. Other Programs

One of the main chosen tools, InterProScan, is actually a portmanteau of several applications and reference datasets (PFAM, PANTHER, etc.), that provide essential domain and functional site information on protein sequences. Moreover, most of the retrieved matches normally provide GO code correspondences that can be summed up to those coming from other methods used in the pipeline.

SignalP and targetP as standalone programs [43] and CD-search [44] as an executed web service also provide sequence, domain and cellular information. However, at the time of writing, they did not deliver the GO mappings.

### 2.7. Integration and Reports

The results of the different analysis steps described above are uploaded into a central database that already keeps track of the sequence identifiers uploaded during the preprocessing part. 

Pipeline output and report files are stored in a user-defined results directory, being placed in that location as soon as involved processes are completed. This allows users to inspect the pipeline progress and to stop it and resume it at any moment, if needed. 

As last steps of the pipeline, a summary text file (total_stats.txt) and an image PNG file (annotatedVsnot.png) are generated with some statistics and the coverage of the annotation. These are produced along with the GFF file (its name is the same as the one chosen for the database in the configuration file) with protein information on all the matches and functional assignments. Text tab-separated files of GO matches by protein (go_terms.tsv) and by gene (go_terms_byGene.tsv), along with related source methods used from the previous stage of the pipeline, are also available, thus enabling the user to perform subsequent GO-based enrichment analyses [45].

Moreover, other convenient TSV files are also produced with extensive detail of the matches from the involved application (interProScan.res.tsv, signalP.res.tsv and targetP.res.tsv). Likewise, a protein_definition.tsv file is provided with suggested descriptions of candidate proteins, normally derived from the first hits of BLAST applications, which can be convenient for rapid inspection by curators. 

In addition, different files from the GFF cleaning and verification preprocessing part of the pipeline can be found in the same location, that is, the cleaned GFF input file (annot.gff), a log of the cleaning process (annot.gff.clean.txt) and some stats associated with it (annot.gff.stats.txt).

## 3. Results

### 3.1. Running FA-nf

Next, we detail the steps to run the pipeline.

- Ensure you have a recent version of Git software and clone the FA-nf repository. This will create a FA-nf folder with the pipeline contents.
  ○ $ git clone --recursive https://github.com/guigolab/FA-nf
- You can otherwise download and extract a specific release version from the following:
  ○ https://github.com/guigolab/FA-nf/releases
- Ensure you have either Docker (at least 19.x version) or, preferably, Singularity (at least 3.2.x version) installed.
  ○ Docker installation details: https://docs.docker.com/install/ (accessed on 19 October 2021).
  ○ Singularity installation details: https://singularity.hpcng.org/admin-docs/3.7/installation.html (accessed on 19 October 2021).
- Install Nextflow (version 20.10.0 tested). In this example, we keep it in the same directory as the pipeline. Otherwise, you would normally place it somewhere in the PATH of your system. Java 8 or later must be available in the system.
  ○ $ cd FA-nf; export NXF_VER=20.10.0; curl -s https://get.nextflow.io | bash
- If you plan to use Interproscan with private software, follow the container image generation instructions that can be found under the containers/interproscan directory of the repository.
- If you want to use privative programs, such as signalP and targetP, prepare a container image following the instructions under the containers/sigtarp directory of the repository. Otherwise, the execution of these applications can be skipped from the pipeline configuration.
- Optionally, you also can set up your custom GOGOApi REST API service from the instructions provided under the gogoapi directory of the repository.
- If your system does not have internet connection, you can generate Singularity files in advance and modify accordingly the container tag values in the nextflow.config file. Some pregenerated Singularity container images can be found at https://biocore.crg.eu/containers/FA-nf/ (accessed on 19 October 2021).
- Download (and index when necessary) all the datasets used by the pipeline, as detailed in the repository documentation. At least some BLAST, Interproscan and KofamKOALA files are needed.
  ○ A Nextflow pipeline script for downloading necessary datasets (download.nf) is available. A sample configuration file (params.download.config) is available for convenience. The datasets will be downloaded and indexed using the following command:
    ■ $ ./nextflow run -bg download.nf --config params.download.config &> download.logfile
  ○ Alternately, some convenient scripts for setting and indexing the necessary datasets can be found at https://github.com/toniher/biomirror/
  ○ As a last instance, some minimal test datasets can be found here: https://biocore.crg.eu/papers/FA-nf-2021/datasets/ (accessed on 19 October 2021).
- For sake of information, we provide some indicative space usage numbers below.
  ○ NCBI databases (update_blastdb.pl) (nr, 349 G index size)
    ■ Formatting with Diamond (nr, 187 G index size)
  ○ Interproscan (5.48-83.0, 89 G)
  ○ KofamKOALA ftp://ftp.genome.jp/pub/db/kofam/ (202103 > ko_list, profiles, KO text files, 13.5 G)
  ○ Datasets used for GOGOApi retrieval service.
    ■ UniProt ID mapping (89 G uncompressed)
    ■ GOA. Uniprot proteins and GO accession codes mapping (11 G compressed)
    ■ Final database size: ~250 G
- Check params.config file and adapt its values to your system configuration and to work with your input files and datasets locations as defined in previous points
- Check nextflow.config file and adjust it according to the characteristics of your HPC queue system by replacing queue names and increasing or decreasing aspects such as CPU or RAM. More details at: https://www.nextflow.io/docs/latest/config.html
- You can start the execution of the pipeline (normally from the node with access to an HPC queue system) with:
  ○ $ ./nextflow run -bg main.nf --config params.config &> logfile
- We can check how the pipeline is progressing with:
  ○ $ tail -f logfile
- As the pipeline advances, intermediary and final results are stored in resultPath directory, as defined in params.config file. More details can be found in the README file of the software repository.

### 3.2. Example Cases

**Figure 3:**
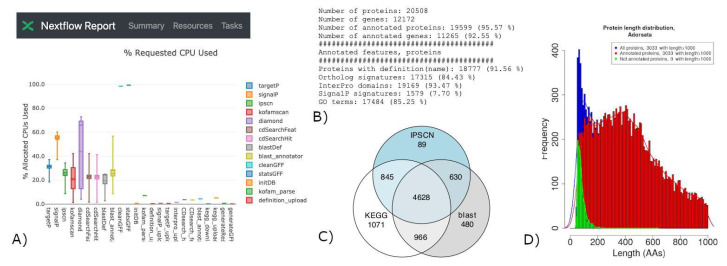
FA-nf annotation run results on *Apis dorsata*. (**A**) Example of NF report; (**B**) example of annotation summary; (**C**) proteins annotated with GO terms by annotation sources; (**D**) length distributions for annotated and unannotated proteins.

As a first example, FA-nf was run against the gene structural annotation of *Apis dorsata* data [46], consisting of 20,508 translated protein sequences from 12,172 associated genes described in a FASTA and a GFF file, respectively. The pipeline was run using the MySQL engine, opting out external web-based annotation services, against the NCBI NR database (202,010) formatted for DIAMOND in default mode, using the *E*-value threshold of 1 × 10^−5^, InterproScan 5.48-83, and KOFAMscan with the 202103 dataset. It took less than 8 h to run the pipeline on the cluster, for a total of 815 CPU hours. The functional annotation was obtained for 19,599 proteins (95.57%) and 11,265 associated genes (92.55%). A total of 17,475 GO terms could be retrieved with the “common” BLAST annotator service strict method, and up to 17,540 with both “most” and “all” less conservative approaches. It is worth noting that different tests using NCBI-BLAST+ with the same parameters took considerably longer times, up to 2 days, with 19,638 (95.76%), with an annotation of 11,304 (92.87%) proteins and genes, respectively, and up to 17,636 GO terms retrieved with the BLAST annotator “all” mode (Figure 3).

In another case, using the same configuration parameters and datasets with DIAMOND, a genome annotation of *Phaseolus vulgaris* [47], consisting of 57,327 proteins and 41,885 genes, took around 1 day and 1604 CPU hours. A total of 54,878 proteins (95.73%) and 39,506 genes (94.32%) were annotated with at least one application. A total of 45,428 and 47,279 GO terms could be retrieved with the BLAST annotator service “common” and “all” modes, respectively.

As a last independent example, the pipeline was also run with the same parameters as described above with DIAMOND and the “all” BLAST annotator mode against the NCBI available *Abscondita terminalis* genome (https://www.ncbi.nlm.nih.gov/genome/84492) (accessed on 19 October 2021) [48]. From 20,439 proteins, 20,434 (99.98%) could be annotated with at least one method and 15,628 GO terms could be retrieved.

Input, output and configuration files of these examples are linked in the README file of the “dataset” folder from the pipeline repository.

## 4. Discussion

Functional annotation provides two main outcomes: one is the functional elements assigned to genes, and another one is an additional quality check of the genome assembly and structural annotation. Assigning functional elements—functional domain, GO terms, metabolic pathways, and others—allows downstream analysis of specific genome properties. At the same time, the presence of genes belonging to a different kingdom may indicate contamination from the upstream assembly [49], and the presence of particular domains or homology hits may help to separate transposable elements from protein-coding genes [50].

On the other hand, the choice of tools in this process may also pose a dilemma between sensitivity, that is, how many new sequences are annotated, and the time and computing resources the user is willing to invest to retrieve some additional annotated sequences, compared to a faster method that might end up not spotting them. This is actually the case when searching for orthologs and, accordingly, with annotation processes based on orthology methods [51].

Another concern for any functional annotation approach is the bias against annotating short sequences [52]. As it seems, short peptides, even those normally assigned to non-coding regions [53], might be actually transcribed and translated, thus eluding their detection in structural and functional pipelines. This might eventually improve when source databases are updated, but it could also require including additional tools apart from the ones currently considered in the presented pipeline. We need to emphasize, in any case, that FA-nf aims to functionally annotate protein coding genes, and it cannot be used to annotate long or short non-coding RNAs. Since these usually lack strong homologues with characterized functions in other species, their functional annotation by computational means is extremely challenging, and the approach in FA-nf cannot be employed here.

On the other hand, it is worth recalling that one of the present-day major concerns in research revolves around the reproducibility of published experiments. This also applies to bioinformatics and computational analyses [54]. In these fields, reproducibility must be considered at the level of both data and code [55]. Since functional analyses are dependent on the state of used databases at the time of the execution, care should be taken to preserve these datasets along with input and result files [56].

As a strong point of FA-nf, if the involved datasets are stored, preserving versions or timestamps as presented in previous sections, it becomes possible to reproduce previous analyses and compare them to newer ones. This is relevant for the datasets that their providers are continuously updating, but not for keeping discrete releases, such as the NCBI BLAST ones. The only exception would be for data retrieved by using web services, such as CD-Search [44], which can be skipped anyway if desired. Moreover, data obtained from analyses can always be reused from the FA-nf database as text dumps. A possibility for helping users to share input, configuration and output data could be integrating programmatic access to public repositories such as Zenodo [57], or data management systems such as Datalad [58].

More specifically, at the level of code, different release versions of the pipeline are kept in the Git repository and archived automatically in Zenodo when published. Moreover, software is encapsulated within containers and, if using Singularity, users may even decide to store software image files along with the code. Indeed, with Nextflow, it is possible to define a specific release of its engine with the NXF_VER environment variable. Thanks to that, different versions of the pipeline would continue working, even if non-backwards compatible syntax changes are introduced in future versions of Nextflow software. This enables users to compare and evaluate results based on different pipeline and included software container versions.

## Figures and Tables

**Table 1:** Non-exhaustive summary table of existing functional annotation programs or pipelines.

Program/Pipeline	Installation	Used Software	Datasets	Comments
Blast2GO [20]	Local installation and web/cloud services	BLAST+, Interproscan, BLAST2GO specific software, etc.	Custom, Normally, NCBI BLAST DBs, InterPro, GO	Subscription tool. Visualization dashboard. Gene structural annotation options. Newer versions integrated into other toolboxes.
eggNOG mapper [9]	Web service and local installation	DIAMOND, HMMER	eggNOGdb (from several sources), GO, PFAM, SMART, COG	Available command-line tool and REST API for querying the service. Gene structural annotation options.
FA-nf	Local installation	BLAST+, DIAMOND, Interproscan, KOFAM, CDD, SignalP, TargetP, etc.	Custom. Normally, NCBI BLAST DBs, InterPro and UniProt-GOA	Based on Nextflow pipeline framework and software containers.
GenSAS [15]	Web service	BLAST+, DIAMOND, Interproscan, SignalP, TargetP, etc.	SwissProt/TrEMBL, RefSeq, RepBase	No installation needed. Requires web user registration. Includes gene structural annotation and visualization. There can be resources and usage restrictions.
MicrobeAnnotator [18]	Local installation	BLAST+, DIAMOND, KOFAM.	SwissProt/TrEMBL, RefSeq, KEGG	Focused on microbiomes. Conda/Python based.
PANNZER2 [17]	Web service	SANSparallel	UniProt, UniProt-GOA, GO, KEGG	Available command-line tool for querying the service.
Sma3s [19]	Local installation	BLAST+	Reference datasets generated from UniProt, GO	A Perl script. Simple installation.

## Data Availability

Example datasets and results can be found at: https://github.com/guigolab/FA-nf/tree/master/dataset (accessed at 19 October 2021).
